# Antibacterial Effects of the Novel Blue Laser and Erbium Chromium Laser on *Enterococcus* Faecalis in Root Canal Dentin with Different Thicknesses

**DOI:** 10.1155/2022/6119464

**Published:** 2022-03-25

**Authors:** Seyedeh Sareh Hendi, Erfan Ahmadyani, Mohammad Yousef Alikhani, Maryam Farhadian, Alireza Mirzaei

**Affiliations:** ^1^Faculty of Dentistry, Department of Endodontics, Hamadan University of Medical Sciences, Hamadan, Iran; ^2^Faculty of Medicine, Department of Microbiology, Hamadan University of Medical Sciences, Hamadan, Iran; ^3^Department of Biostatics, School of Public Health and Research Center for Health Sciences, Hamadan University of Medical Sciences, Hamadan, Iran; ^4^Faculty of Dentistry, Department of Laser Research, Islamic Azad University, Tehran, Iran

## Abstract

The aim of this study was to investigate the antibacterial effects of 445 nm blue diode lasers and erbium chromium lasers on the biofilm of *Enterococcus faecalis* in root canal dentin with different thicknesses. Dentin slices with thicknesses of 300, 500, and 1,000 microns were prepared; and after the biofilm formation, they were randomly divided into four groups: group A : Er, Cr:YSGG laser radiation; group B: 445 nm diode laser radiation; group C : laser radiation in three cycles; and control group D: 5 samples of each thickness were selected as a positive control group. Er, Cr:YSGG and diode lasers alone did not significantly reduce the number of bacteria in any of the thicknesses. Only in 1 mm thick sections, the group exposed with the both Er, Cr:YSGG laser and 445 nm diode laser (66%) significantly reduced the number of bacteria. There was a significant difference in a thickness of 0.3 mm compared to a thickness of 1 mm, indicating that these lasers had a better effect at a thickness of 0.3 mm than at 1 mm parts (*P*-value < 0.008). It seems that these lasers can be used as an adjunct to conventional chemical methods in cleaning infected canals.

## 1. Introduction

Since maximum removal of bacteria from the root canal is one of the most important concerns in the endodontic treatment, mechanical preparation and chemical disinfection of the root canal system are the most important steps in the endodontic treatment [1, 2]. The anatomical complexities in the root canal system, such as the presence of isthmus, accessory canals, and dentinal tubules, are the site of bacterial survival after routine canal cleansing [3]. For this reason, the mechanical instrumentation alone is not able to completely eliminate the bacteria, so different chemicals and disinfection techniques are needed to reduce the number of bacteria [4]. Mechanical cleaning includes the removal of debris along with canal sliding. Chemical debridement is also effective in removing residual tissues and biofilm of bacteria from parts of the canal that did not undergo mechanical debridement. In general, root canal therapy is a procedure for minimizing the pathological factors in the root canal system. Therefore, the main goal of endodontic treatment is to prevent the development or disappearance of apical periodontitis [5, 6]. However, in some cases, the root canal treatment is likely to fail for a variety of reasons, including the complexities of the root canal structure and the bacterial resistance [7–9].

*Enterococcus faecalis* is a Gram-positive anaerobic bacterium and is the most common species found in secondary endodontic infections [10]. This bacterium is resistant to antibiotics and can survive in adverse nutritional conditions inside the canal. It also has the ability to form biofilms and penetrate into dentinal tubules. Bacterial biofilms are highly resistant to detergents due to the formation of extracellular polymer matrix [7, 8]. For these reasons, new alternative disinfection techniques are needed to advance canal disinfection. Among various canal cleaners, the most important are sodium hypochlorite with tissue solubility and maximum antibacterial power to combat these contaminants. However, sodium hypochlorite penetrates into the dentinal tubules only 130 microns due to the low surface tension. However, bacteria can penetrate into the tubules to a depth of 1,000 microns [8].

Recent studies have shown the effects of laser in canal disinfection. Among the types of lasers, diode lasers have been widely used in dentistry due to their small sizes, low prices, and easy operations [11–13]. Diode lasers have little interference with water and hydroxyapatite due to their flexible fibers, which can easily penetrate into curved canals [14]. Examining the effects of a 980 nm diode laser on reducing the biofilm of Enterococcus *faecalis* bacteria in a canal, Prazmo et al. [15] reported that the number of bacteria significantly decreased in this way. Kerstin Bitter in 2017 also compared the antibacterial effects of ozone gas and a 980 nm diode laser with the antibacterial effects of a canal coated with calcium hydroxide and chlorhexidine gel for one week. Their results showed that the antibacterial effects of ozone gas and 980 diode laser were similar to those of one-week use of intracanal drugs [16]. Recently, a 445 nm blue laser device has been introduced for dental applications such as reducing the number of bacteria, blood coagulation, and soft tissue incision. Blue laser is a laser that emits electromagnetic radiation with wavelengths of 360 to 480 nm, and the human eye sees it as blue or purple [17]. Compared to the red light systems, one of the most important advantages of blue laser light is that due to its shorter wavelength and higher absorption in hemoglobin and melanin chromophores, it spreads less and penetrates into lesser depth in the tissue. For this reason, this method reduces damage to deep layers and is, therefore, more accurate than other methods [18].

Erbium laser is one of the lasers used for endodontic treatment. The bactericidal effects of this laser have been proven in various studies. In addition, the bactericidal effects of this laser show minimal thermal damage. The effects of erbium laser on *Enterococcus faecalis* were confirmed by the studies conducted by Perin et al. [19] and Noiri et al. [20]. The results of Kuvvetli's et al. study [21] on permanent teeth showed a stronger bactericidal effect of erbium laser compared to NaOCl. Due to the novelty of this method and the lack of sufficient information on the use of 445 diode lasers and its success rate in the endodontic treatment, it seems that further studies are essential in this field and on the simultaneous effects of this laser and erbium laser and on the effectiveness of these lasers in different dentinal thicknesses. The bactericidal effects of 445 nm diode laser and erbium laser have not been compared, and this is the first research that has examined the simultaneous effect of these two lasers. So the aim of this study was to evaluate the antibacterial effects of 445 nm diode laser and Er, Cr:YSGG laser and simultaneous effects of them on the *Enterococcus faecalis* biofilm in dentinal tubules of different thicknesses.

## 2. Materials and Methods

**Figure 1:**
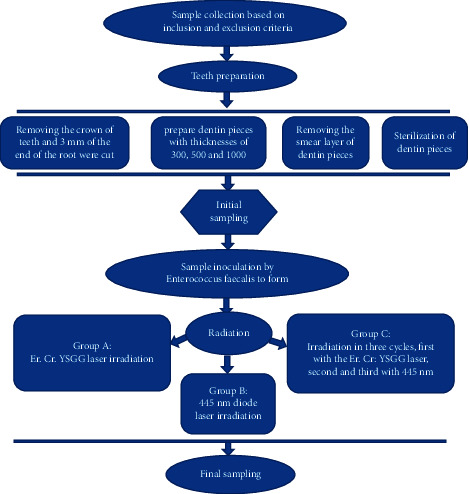
Flowchart for design study.

This experimental laboratory study was performed on 60 human single-rooted teeth (central maxilla or canine of both jaws, mature and without symptoms of root resorption) in the endodontics department at the School of Dentistry in Hamedan University of Medical Sciences. The flowchart is shown in Figure 1.

### 2.1. Materials

Diamond milling (Tees Kavan, Iran) was used to remove the crown and end of the root.

Normal saline (sodium chloride, 0.9%) (Shahid Ghazi co., Tabriz, Iran) and 5% sodium hypochlorite (Paxan Co., Tehran, Iran) were used for irrigation of teeth.

EDTA 17% (Maraboon, Iran) was used to remove the smear layer.

The standard strain of *E. faecalis* (ATCC29212), trypticase soy broth, TSB (HiMedia Laboratories, Mumbai, India), PBS (phosphate-buffered saline), sheep blood agar plates (HiMedia Laboratories, Mumbai, India), and Eppendorf tube (Amin Co., Iran) were used for microbiologic experiments.

A microtome device (Nemo, Iran) was used to cut teeth.

The 445 nm diode laser (SiroLaser Blue, Sirona Dental System GmbH, Germany) and erbium chromium laser (Biolase, Waterlase/USA) were used for radiation.

### 2.2. Inclusion and Exclusion Criteria

Inclusion criterion included freshly extracted anterior single canal teeth confirmed by radiography as a single canal. Exclusion criteria included multirooted teeth, teeth with any internal or external decay in the root, the presence of developmental defects, and teeth with caries in the root surface.

### 2.3. Teeth Preparation

Hard and soft tissues adhering to the teeth were removed using an ultrasonic scaler after immersing the samples in 1% hypochlorite for 24 hours. The teeth were rinsed with tap water and kept in normal saline. Then, the crown of the teeth and 3 mm of the end of the root were cut by the use of a diamond mill (Tees Kavan, Iran) to build dentin blocks of 10 mm long. Then, the dentin blocks were longitudinally cut using a microtome (Nemo, Iran) to prepare dentin pieces with thicknesses of 300, 500, and 1,000 microns. The thicknesses of dentin parts were controlled in a Vernier device, and parts with a tolerance of less than 5 lm were excluded. A total of 105 pieces of dentins were immersed in an ultrasonic bath containing ethylenediaminetetraacetic acid 17% (EDTA 17%) for 4 minutes in order to remove the smear layer, were washed with normal saline, and then maintained in a saline solution at 4°C until the next step. The dentinal tubules were autoclaved at 151°C for 15 minutes. To evaluate the accuracy of sterilization, a control group consisting of 10 dentin pieces was considered. The sterilized parts were washed with 0.09% sterile sodium chloride. The liquid was poured into a blood agar plate, and after 24 hours of incubation, no bacterial growth was confirmed.

### 2.4. Bacterial Inoculation

Standard species of *Enterococcus faecalis* (ATCC 29212) were cultured in the brain-heart infusion (BHI) broth and were incubated for 24 hours at 37°C. One hundred ml of sterile BHI medium and 30 *μ*l of suspension containing 10^9^ CFU/mL of *Enterococcus faecalis* were added to dentin pieces. In order to form a biofilm, the samples were incubated at 37°C for 4 weeks. Every two days, 30 *μ*l of BHI solution was replaced with fresh sterile BHI medium under sterile conditions.

### 2.5. Radiation

Dentin slices with different thicknesses were randomly divided into three groups: group A: including 10 pieces of each thickness of samples (30 pieces) exposed to Er, Cr:YSGG laser irradiation (1.5 W, 50 Hz, 50 *μ*s pulse duration, 60% water, and 30% air pressure) based on Ramalho's et al. [19] and Esteves Oliveira's et al. [23] approaches. The laser irradiation in one cycle consisted of three stages of 10-second irradiation with a 5-second interval between them for a total irradiation time of 30 seconds. We used the RFTP5 tip for Er, Cr:YSGG radiation.

(1) $$\begin{array}{c} E_{\text{density per pulse}}:\frac{E_{\text{per pulse}}}{A},\\ E_{\text{per pulse}}=P_{\text{peak}}\times \text{Pulse duration,}\\ =600_{w}\times 50\times 10^{-6}=0.3_{J}\\ E_{\text{density per pulse}}=\frac{0.3}{0.002}≅150\frac{J}{cm^{2}}. \end{array}$$

Group B: 10 pieces of each sample thickness (30 pieces) were exposed to 445 nm diode laser (SiroLaser Blue, Sirona Dental System GmbH, Germany). The laser irradiation was performed through a tip with a diameter of 200 *μ*m (200-*μ*m-diameter EasyTip, REF 6535905). The stability of the output power of the device was confirmed using a power meter (FieldMax-Top, Coherent Inc., CAWA). During one cycle, there were three stages of 10-second irradiations with a 5-second interval between them for a total irradiation time of 30 seconds.

(2) $$\begin{array}{c} E_{\text{density per second}}=2000\frac{J}{cm^{2}},\\ E_{\text{density per total}}=540\frac{J}{cm^{2}}. \end{array}$$

Group C: 10 pieces of each sample thickness (30 pieces) were exposed to the laser irradiation in three cycles including nine 10-second irradiations. In the first cycle, the samples were irradiated with the Er, Cr : YSGG laser with the same power settings as in group A. The second and third cycles of radiations both simultaneously included the first cycle with the 445 nm diode laser (0.6 W, CW 200 µm fiber), and the total radiation time was 90 seconds for group B. The control group D: 5 samples of each thickness (15 pieces) were selected as the positive control group (without treatment), which was not laser irradiated. The pieces were irradiated from the opposite side of the bacterial inoculation. This indirect radiation was used to evaluate the effects of the dentin thickness on the bactericidal effects of the two laser wavelengths. In order to simulate the radiation inside the root canal, the pieces were irradiated parallel to the tooth surface in a spiral or rotational motion in close association with a 1 mm/sec scanning pattern, and there was a 5° incident angle between the fiber tip and the dentinal piece.

### 2.6. Initial Sampling

A sample of the microbial suspension was taken before irradiation and disinfection of the components and transferred to a sterile microtubule (Amin Co., Iran) containing 1 ml of normal saline solution (0.9% NaCl), was vortexed for 20 seconds, and was serially diluted 10 times to prepare a concentration of 0.01. Finally, 100 *μ*l of each solution was dispersed on a sheep blood agar (Merck Co., Germany) plate and incubated for 24 hours at 37°C. The number of bacterial colonies was counted according to the CFU/mL.

### 2.7. Final Sampling

To standardize all the groups, the dentinal tubules were washed with 5 ml sterile saline and left for 30 seconds. Under sterile conditions, the sample was transferred to the microtubes containing 1 ml of the saline solution and was vortexed for 20 seconds, and counting of the colony units was performed as in the initial sampling.

### 2.8. Statistical Analysis

The data analysis was performed using SPSS software version 21. The descriptive statistics methods and statistical tests such as the one-way analysis of variance (ANOVA) and *t*-test were used to analyze the data, and the nonparametric tests were used if the normality hypothesis was not established. The significance level in all the tests was considered to be 0.05.

## 3. Results

**Figure 2:**
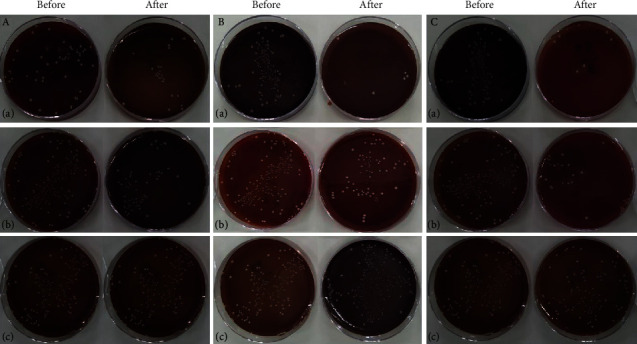
Bacterial culturing before and after irradiation. (a) Er, Cr:YSGG laser, (a) 0.3 mm thickness; (b) 0.5 mm thickness; and (c) 1 mm thickness. (b) 445 nm diode laser: (a) 0.3 mm thickness; (b) 0.5 mm thickness; and (c) 1 mm thickness. (c) Er, Cr:YSGG and 445 nm diode lasers, (a) 0.3 mm thickness; (b) 0.5 mm thickness; and (c) 1 mm thickness.

The results of this study showed that in all three thicknesses of 0.3, 0.5, and 1 mm, the lowest number of the remaining bacteria belonged to the group that was simultaneously affected by the two types of Er, Cr:YSGG and 445 diode lasers. The percentages of the bacterial reduction for each group were obtained using the difference between the control group and each group divided by the number of bacteria in the control group (Table 1, Figure 2).

Kruskal-Wallis (KW) test with a significance level of less than 0.05 was used to evaluate the effects of the lasers in different thicknesses of dentinal tubules. Since the only group with a significant difference was the group with a thickness of 1 millimeter (Table 2), the groups were compared in pairs for this thickness.

We compared the groups by pairwise comparison in 1 mm thickness using the Mann-Whitney test with Bonferroni correction with a significance level of less than 0.006. According to the defined level of significance, a significant difference was observed in the two groups of Er, Cr:YSGG laser +445 nm diode lasers vs. 445 nm diode laser and Er, Cr:YSGG +445 nm diode laser vs. control group. The results showed that the Er, Cr:YSGG laser +445 nm diode laser group had a greater effect on the bacteria compared to the 445 nm diode laser (*P*-Value < 0.006, Table 2).

Then, in order to evaluate and compare the number of bacteria in different thicknesses for each group, the Kruskal-Wallis test was used with a significance level of less than 0.05 (Table 3). Only a significant difference was observed in the two groups of Er, Cr:YSGG and 445 nm diode lasers (*P*-Value < 0.05). The thicknesses were compared in pairs because the numbers of the bacteria in different thicknesses in the two groups of Er, Cr:YSGG laser and 445 nm diode lasers were significantly different.

**Figure 3:**
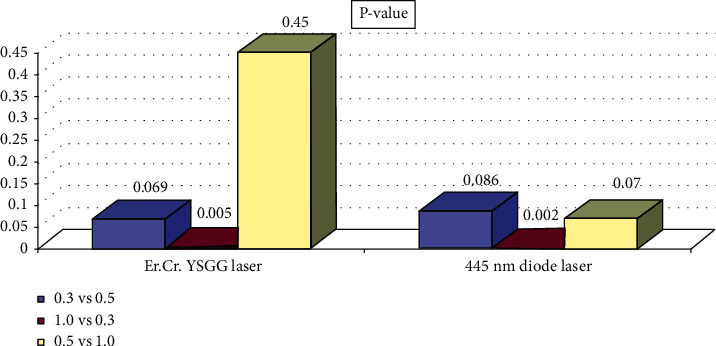
Pair comparison of thickness in two groups of Er, Cr:YSGG and 445 nm diode lasers.

The comparison between the two groups of Er, Cr:YSGG laser and 445 nm diode laser was performed using the Mann-Whitney test and considering a *P*-value less than 0.008 as a significant level (Table 4). In the both groups of lasers, thicknesses of 0.3 mm were significantly different compared to those of 1 mm (*P*-value < 0.008) Figure 3.

## 4. Discussion

Due to the infiltration of bacteria into the dentinal tubules, the depth of the dentinal tubules cannot be properly cleared by the conventional methods [24, 25]. Therefore, to investigate the effects of dentin thickness on the bactericidal effects of the lasers, unlike previous studies in which the bacterial suspension was directly inoculated into the root canal and the laser fiber tip was in direct contact with the bacteria inside the dental canal [26], in this study, the dentinal sections with thicknesses of 300, 500, and 1,000 *μ*m were prepared and indirect laser irradiation was used, i.e., the laser fiber was placed on the opposite side of the bacterial suspension. Thus, the distances between the lasers and the bacteria were 300, 500, and 1,000 micrometers.

The results showed that the Er, Cr:YSGG and diode lasers alone did not significantly reduce the number of bacteria in any of the studied thicknesses. Only in the 1 mm thick sections, the group irradiated with both the Er, Cr:YSGG and diode lasers (66%) significantly reduced the number of bacteria compared to the diode-irradiated group (0%). This finding showed that the simultaneous effect of the two lasers was better than the effect of using only the 445 nm diode laser. In comparison between the groups in terms of the changes in thicknesses, the 445 nm diode laser and the Er, Cr:YSGG laser in sections with a thickness of 0.3 mm were significantly different compared to the case with a thickness of 1 mm, indicating a better effect of these lasers in a thickness of 0.3 mm than in a thickness of mm. The general result of this study, aiming to investigate the antibacterial effect of the 445 nm diode laser and the Er, Cr:YSGG laser on *Enterococcus faecalis* biofilm in the dentinal tubules of different thicknesses, shows a significant effect in the case of separately using the two lasers in thicknesses of 0.3 mm and 1 mm dentin.

In a similar study in 2018, Norbert Gutknecht et al. [24] showed that in continuous radiation using a power of 0.6 W, 81.42%, 83.75%, and 77.42% of bacteria reductions were observed in thicknesses of 0.3, 0.5, and 1 mm, respectively. In our study, the bacterial reductions were 48% and 20% and zero for thicknesses of 0.3, 0.5, and 1 mm, respectively. The differences in results were probably due to the differences in the methods and timing of the laser uses. As in the above study, the irradiation time was longer than in our study, eventually leading to a high energy transfer at 445 nm in hydroxyapatite crystals. In their study, radiations were performed four times, each time for 10 seconds, with a 10-second rest period between the two radiation intervals. In this study, radiation was performed at an angle of about 5° under constant sinusoidal movements. A computer-controlled desk was used to create the same radiation conditions. This device allows the slices to move in a constant sinusoidal motion at a constant speed during irradiation. This computer-controlled desk was also used to fix the specimens during the irradiation, controlling the same laser radiation along with a sinusoidal 0, wave with a moving lateral component. In our study, the samples were fixed by hand and the laser radiation was in the form of a continuous wave, which was different from the above study and probably caused differences in the results of the two studies.

The results of another study by Franzen et al. [27] showed that erbium, chromium : yttrium-scandium-gallium-garnet (Er, Cr : YSGG) laser radiation with a wavelength of 2,780 nm and a power of 0.25 W led to 82% and 58% reductions in the number of bacteria at thicknesses of 300 and 500 *μ*m, respectively. Consistent with this study, the results of their study showed that the Er, Cr:YSGG laser has a significant effect on *Enterococcus faecalis* at a thickness of 300 microns compared to higher thicknesses. The Er, Cr:YSGG laser, on the other hand, could penetrate to a depth of 300 microns; however, in comparing the same thicknesses, the difference in the effectiveness of the Er, Cr:YSGG laser in our study and that study is probably due to the differences in times and powers used. Another major difference was in the formation or nonformation of the biofilm. In this study, radiation mainly occurred as soon as the microorganism was replaced and not after biofilm formation. The irradiation time was 40 seconds (in 4 irradiations).

In another study, Gutknecht et al. [28] evaluated the antibacterial effects of an 810 nm diode laser on *Enterococcus faecalis* in the dentinal parts of bovine teeth with thicknesses of 300, 500, and 1,000 microns. The results of their study showed that the reductions in bacteria were 88.38% and 73.96% at thicknesses of 300 and 500 microns, respectively. In our study, the bactericidal effects at a thickness of 0.3 mm were significantly greater than at a thickness of 0.5 mm (48% and 20%, respectively). Despite the similarity in the methods, the two studies differed in the effects of the laser on the reduction in *Enterococcus faecalis*. This difference was probably due to the bacteria colonization and radiation methods.

Ewa Joana Prazma et al. [15] studied the effects of a 380 W- 980 nm diode laser on the reduction in the intracanal biofilm of *Enterococcus faecalis* and concluded that the diode laser had a significant antibacterial effect and approximately showed a decrease in 87.6% in the double radiation group and 52.5% in the single radiation group. Their results are in line with the results of our study, showing that the use of the diode laser can reduce the number of bacteria. However, due to the differences in the mechanism of action of the two types of lasers (445 and 980 diodes), the wavelengths of the lasers and the irradiation time and the power of the laser used, it was expected that there were differences in terms of their effectiveness. The 980 nm diode laser used in their study had a power of 3 W, which was much higher than the power used in our study. The 980 nm laser was also used in two modes: the first one was a radiation cycle of 1 time and 20 seconds, and the other was a repetition of radiation for 20 seconds with an interval of one minute. Compared to our study, the first case took less time, and in the case of repeated radiation, the radiation time was longer. Research has also shown that there is a possibility of morphological changes and a risk of apical leakage in the radiation of a 980 nm diode laser with the mentioned power [29].

Due to its high energy and change in the structure of dentinal tubules, the 980 nm diode laser cannot be used for a long time. In this regard, the results of another study aimed at comparing the antibacterial effectiveness of chemical detergents and the depth of their penetration into dentinal tubules when used alone and when combined with a diode laser with a wavelength of 810 nm and a Er, Cr : YSGG laser showed that the lowest effect in reducing the amount of bacteria was found in the normal saline group, and the Er, Cr : YSGG laser showed the highest reduction in the number of bacterial colonies and the highest penetration depth when used in combination with sodium hypochlorite and chlorhexidine gluconate [30]. Despite the difference in the wavelengths of this laser and the laser used in our study, the results were almost similar to our study; this means that in the above study, the penetration depth for the diode and Er, Cr:YSGG lasers was approximately 0.3 microns. In our study, the effect of Er, Cr:YSGG and 445 nm diode lasers on reducing bacteria at a thickness of 0.3 mm was better than at the other thicknesses (44% and 48%, respectively), which was probably due to the same usage time and power in the two studies.

## 5. Conclusions

Consistent with the results of previous studies, the results of this study showed the effectiveness of the Er, Cr:YSGG and diode lasers in reducing the number of bacteria in the root canal of the infected teeth. These two lasers showed significant differences at thicknesses of 0.3 mm and 1 mm, indicating the effects of these two lasers on reducing the number of bacteria in the infected dentin. Given that the effect of the simultaneous use of two lasers was greater, it seems that these lasers can be used as an adjunct to conventional chemical methods in cleaning infectious canals. Due to the in vitro nature of this study, it is suggested that long-term clinical studies are designed and performed to achieve more definitive results. In addition, it seems that maintaining the natural structure of the root canal and not causing destructive tissue changes and not destructive increases in the temperature inside the root canal should be considered in designing suitable settings with different wavelengths to remove bacteria. It is suggested that the nondestructive effects of laser radiation should be investigated considering the antibacterial effects of different wavelengths.

## Figures and Tables

**Table 1:** Comparison of the number of bacteria in different groups with the same thickness.

Thickness (mm)	Group	Mean	S.D	Average bacterial reduction (%)	*P*-value
0.3	Er, Cr:YSGG laser	216900.00	103530.40	44	0.285
	445 nm diode laser	205000.00	208595.90	48	
	Er, Cr:YSGG +445 nm diode laser	158111.11	135794.37	60	
	Control	390000.00	176776.69		

0.5	Er, Cr:YSGG laser	463500.00	4513383.07	28	0.096
	445 nm diode laser	514100.00	347925.10	20	
	Er, Cr:YSGG +445 nm diode laser	245800.00	189973.56	62	
	Control	637000.00	331911.13		

1.0	Er, Cr:YSGG laser	457600.00	224649.94	34	<0.001
	445 nm diode laser	969000.00	623270.05	0	
	Er, Cr:YSGG +445 nm diode laser	237300.00	158675.10	66	
	Control	691142.85	139632.16		

^*∗*^Kruskal-Wallis test.

**Table 2:** Comparison of the number of bacteria in different groups with a thickness of 1 m.

Thickness (mm)	Group	*P*-value ^*∗*^
1	Er, Cr:YSGG laser compared to 445 nm diode laser	0.038
	Er, Cr:YSGG laser compared to both erbium and 445 nm diode lasers	0.021
	Er, Cr:YSGG laser compared to control group	0.025
	445 nm diode laser compared to both Er, Cr:YSGG and 445 nm diode lasers	0.001
	445 nm diode laser compared to control group	0.558
	Both Er, Cr:YSGG and 445 nm diode lasers compared to the control group	0.001

^*∗*^Mann-Whitney test with Bonferroni correction.

**Table 3:** Comparison of the number of bacteria in different thicknesses for each group.

Group	Thickness	*P*-value
Er, Cr:YSGG laser	0.3	0.019 ^*∗*^
	0.5	
	1.0	
445 nm diode laser	0.3	0.004 ^*∗*^
	0.5	
	1.0	
Er, Cr:YSGG laser +445 nm diode laser	0.3	0.531
	0.5	
	1.0	
Control	0.3	0.212
	0.5	
	1.0	

^*∗*^Kruskal-Wallis test with a significance level of less than 0.05.

**Table 4:** Two-by-two examination of thicknesses in two groups of Er, Cr:YSGG laser and 445 nm diode laser.

Group	Thickness (mm)	*P*-value
	0.3 vs 0.5	0.069
Er, Cr:YSGG laser	1 vs 0.3	0.005
	1 vs 0.5	0.45
	0.5 vs 0.3	0.086
445 nm diode laser	1 vs 0.3	0.002
	1 vs 0.5	0.070

*∗*Mann-Whitney test.

## Data Availability

The data are available from the corresponding authors on reasonable request.
